# Recent Advancements in Bioactive Natural Products and Nanoparticle-Mediated Drug Delivery in Cancer Therapy

**DOI:** 10.3390/ijms27031356

**Published:** 2026-01-29

**Authors:** Sujay Kumar Bhajan, Anup Kumar Bishwas, Basudeb Dutta, Ayon Bala, Nahida Aktary, Sohyun Park, Muntajin Rahman, Min Choi, Jinwon Choi, Salima Akter, Amama Rani, Bonglee Kim

**Affiliations:** 1 Department of Biotechnology and Genetic Engineering, Gopalganj Science and Technology University, Gopalganj 8105, Bangladesh; sujaybge@gstu.edu.bd (S.K.B.); anupkumarbishwas21@gmail.com (A.K.B.);; 2 Department of Pathology, College of Korean Medicine, Kyung Hee University, Seoul 02447, Republic of Koreashpark0912@khu.ac.kr (S.P.); muntajinrahman899@gmail.com (M.R.); 2022310848@khu.ac.kr (J.C.); aktersalima@khu.ac.kr (S.A.); amama.rani@khu.ac.kr (A.R.); 3 Korean Medicine-Based Drug Repositioning Cancer Research Center, College of Korean Medicine, Kyung Hee University, Seoul 02447, Republic of Korea

**Keywords:** bioactive natural products, nanoparticles, cancer therapy, drug delivery, tumor microenvironment

## Abstract

Cancer remains a leading cause of death worldwide, necessitating new treatment strategies. Bioactive natural products are a promising source of anticancer drugs because of their complex structures and ability to target multiple pathways, such as inducing apoptosis and inhibiting angiogenesis. However, issues like poor solubility, stability, and bioavailability have limited their clinical use. Nanoparticle-based drug delivery offers a revolutionary approach by improving the pharmacokinetics and targeted delivery of anticancer agents, including natural compounds. Different types of nanoparticles, such as liposomes, polymeric nanoparticles, and metallic nanoparticles, use the enhanced permeability and retention (EPR) effect to increase drug accumulation in tumors while reducing off-target toxicity. This study explores the potential of combining natural products with nanoparticle delivery systems to overcome drug resistance and enhance therapeutic effectiveness. It highlights significant achievements, including FDA-approved nanoparticle-based drugs and innovative combination therapies. The review also discusses current limitations, such as toxicity, scalability, and regulatory hurdles, while looking ahead to emerging themes like smart nanoparticles and personalized medicine. Ultimately, this review emphasizes the importance of integrating bioactive natural products with nanotechnology, presents future research directions, and advocates for translational studies and clinical trials to advance cancer treatment.

## 1. Introduction

Cancer is one of the most dreadful non-communicable diseases that can drastically deteriorate an individual’s life. One in every six deaths occurs due to cancer, and it continues to haunt mankind with almost 10 million deaths in every year, making it the second leading cause of death worldwide [1]. It is estimated that by 2030, the number of new cancer cases per year will give rise to 23.6 million, and unfortunately, the number is only expected to increase exponentially in the coming years [2]. Cancer research has always been a challenge as its complexity due to several types of cancer may exhibit substantial differences in relation to genetic alterations, organ involvement, prognosis, and therapeutic management [3]. With the passage of time, various treatment methods have evolved as scientific knowledge and technology have advanced [4]. Among them, chemotherapy, radiation therapy, immunotherapy, and surgery are the cancer treatments that are being employed mostly around the world [5].

The decision to use such therapies alone or combined with other treatments depends on the cancer type and stage [6]. Whatever the conventional treatment procedure is, though they have effectiveness on cancer, they always show extreme or limited local or systemic side effects. For instance, chemotherapeutic drugs disrupt the metabolic reactions in normal cells and are responsible for nausea, vomiting, alopecia, neuropathy, and multidrug resistance [4]. On the other hand, single-molecule targeted therapy has fewer adverse effects on normal cells, but it triggers the mechanism responsible for drug resistance along with diarrhea and hypertension [7]. Other conventional therapeutic options are effective against malignant growth, but they have several constraints; for instance, anemia, baldness, recurrence of cancer, and emesis with the symptoms that of cancer patient possesses. Moreover, the heterogeneous nature of cancer has hindered the effectiveness of traditional treatment modalities, including radiation therapy and standard chemotherapy. These approaches lack selectivity, often leading to the destruction of both malignant and healthy cells, which in turn causes severe hematological toxicities and significant tissue damage.

Therefore, efforts are ongoing to find better alternatives that maintain effectiveness while reducing toxicity and following measures to prevent drug resistance [7]. Researchers have begun exploring innovative and new treatment approaches and drug delivery systems to combat cancerous tumors more effectively and efficiently [8]. Consuming natural sources like vegetables, fruits, spices, and grains can help prevent cancer development and progression by inducing apoptosis and regulating the cell cycle [9]. About 35,000 bioactive components, such as terpenoids, volatile oils, phenolics, alkaloids, flavonoids, and others, have been derived from plants, marine sources, and others that help reduce the adverse effects associated with modern treatments like chemotherapy and radiotherapy [8].

Although bioactive natural compounds offer numerous health benefits to cancer patients, they present several limitations. Challenges of using natural compounds in cancer include the following: (1) Low bioavailability, (2) Limited exposure to the target site, (3) Affecting both healthy and cancerous cells, (4) Off-target effects, and (5) Susceptible to degradation. Researchers conclude that the use of nanoparticles for delivering natural compounds addresses these challenges by enhancing bioavailability, enabling targeted delivery, improving stability, ensuring controlled release, and facilitating deeper penetration into tumor tissue [9,10]. While the research is ongoing and inconclusive, the utilization of nanoparticle-based natural compounds presents a promising potential therapeutic approach for cancer treatment [9,11]. This article provides an overview of using natural compounds as therapeutic agents for cancer treatment, their effects on cancer cells, and the limitations of this approach alone. Subsequently, this will introduce nanoparticle-based drug delivery, with a focus on natural compound nanoparticles, highlighting their benefits, limitations, and challenges to pave the way for future studies.

## 2. Bioactive Natural Products in Cancer Therapy

Bioactive natural products and their derivatives have exhibited significant potential in the advancement of chemotherapeutic agents, characterized by extensive structural, molecular, and pharmacological diversity [12]. In the 1980s, 1990s, and 2000s, 49% of the total anticancer molecules were from natural products or their derivatives. Additionally, several plant-based antitumor drugs are currently in clinical trials, including taxanes, vincristine, vinblastine, and podophyllotoxin analogs. Moreover, aclitaxel, podophyllotoxin, docetaxel, analogs, and etoposide also show promising anticancer activities [13,14].

### 2.1. Overview of Natural Products

Natural products are substances derived naturally from any living organism, such as animals, plants, insects, marine organisms, and microbes. They are the precious gifts of nature possessing pharmacological and biological properties with an irreplaceable role in the design and development of many drugs. Since ancient times, various natural products (Table 1) have been used to treat many diseases. Even dental plaques reveal that Neanderthals already used plants for medicinal purposes approximately 50,000 years ago [15]. Ongoing research indicates that nature-derived products have the potential in drug design against cancer [16].

### 2.2. Historical Use in Cancer Treatment

Nearly 80% of the world’s population depends on natural sources, mainly plants, microbes, and marine-based products [28]. Since cancer is a rapidly growing and devastating disease, the use of natural products in cancer treatment is highly valuable. However, the modern understanding of cancer differs greatly from ancient times. The word ‘cancer’ comes from the Greek word Karkinos, which originally referred to tumors. Our current knowledge of cancer and its treatments has developed only in recent decades. Over the past 100 years, there has been a revolution in our understanding of this disease. Many natural compounds have been identified as effective agents against cancer [18]. For example, one of the most significant discoveries is paclitaxel (previously Taxol), a well-known compound used to treat breast and ovarian cancers worldwide, discovered in 1962 by the US Department of Agriculture. Green tea antioxidant EGCG (epigallocatechin-3-gallate) has recently been shown to significantly slow the progression of breast cancer in female mice [19]. The discovery of early antibacterial agents between the 1920s and 1940s prompted extensive research into finding anticancer compounds derived from microorganisms. Subsequently, Selman Waksal and H. Boyd Woodruff created the first anti-tumor antibiotic in 1940 from actinomycin D, derived from Actinomyces antibioticus, which acts as an RNA polymerase inhibitor. Anthracycline glycosides and daunomycin, which possess stronger antileukemic properties, were derived from Streptomyces species and gained attention in the 1950s and 1960s [20]. Oceans have been an important source of bioactive natural compounds since the 1950s, but limited technological knowledge has often been a barrier [21]. Nonetheless, the discovery of marine anticancer agents began in 1960, with the development of cytarabine, a pyrimidine nucleoside analogue, which demonstrated promising efficacy against leukemias and lymphomas. During the 1980s and 1990s, other significant bioactive natural compounds with anticancer and antitumor activities (e.g., halicondrins, didemnins, salinosporamide) were isolated and began clinical testing [22]. Natural products have played a crucial role in the history of developing anticancer drugs. In addition to those mentioned earlier, many commonly used cancer treatments are derived from natural sources, including irinotecan, etoposide, and vincristine from plants; actinomycin D and mitomycin C from bacteria; and bleomycin from marine organisms. Several of these compounds remain vital in cancer therapy and are expected to continue playing an important role in the future.

### 2.3. Key Bioactive Natural Products

Bioactive natural products are naturally occurring compounds produced by living organisms, such as plants, microorganisms, and marine life [23]. They influence biological systems through their pharmacological and therapeutic properties. They are enormous in numbers, diverse in chemical and structural characters, and possess a wide range of biological effects. Bioactive natural products can be classified in many ways. For convenience and relevance to the context, this section adheres to the classification of bioactive natural products based on their chemical features, mainly into four groups, namely, polyphenols, alkaloids, terpenoids, and flavonoids.

#### 2.3.1. Alkaloids

Alkaloids refer to the chemical compounds found in plants, animals, and microbes as naturally occurring secondary metabolites containing one or more nitrogen atoms in their cyclic structure [24]. They range from simple and linear to complex and cyclic based on their diversified chemical structures. Though there is no uniform pattern of classifying them, they can be classified into true-, proto, polyamine-, peptide-, cyclopeptide, and pseudoalkaloids. Among the discovered natural products, alkaloids have earned significant attention for cancer treatment due to their capacity to regulate the signaling pathways associated with cancer formation and progression [25]. The PAMT (PI3K/Akt/mTOR) pathway is considered one of the most important signaling cascades in cellular proliferation, survival, and angiogenesis. Therefore, paying attention to the PAMT pathway has been a crucial strategy for treating cancer [26]. Alkaloids, including vincristine, berberine, evodiamine, and vinblastine, among others, have displayed the capacity to selectively affect the PAMT pathway by directly blocking the pathway constituents or disrupting upstream regulators or downstream effectors [25].

#### 2.3.2. Polyphenols

Polyphenols constitute approximately 10,000 phytochemicals characterized by common structural properties, including a three-ring flavan core and multiple phenolic moieties. They are considered the largest group of bioactive phytochemicals and the most extensively distributed across the plant kingdom [27]. Based on their chemical structures, these natural compounds are further classified into several subdivisions such as flavonoids, anthocyanins, catechins, isoflavones, chalcones, curcuminoids, phenolic acids, and others [28]. Various clinical studies demonstrated that one-third of cancer mortality rates are directly correlated with diet, food, and nutrition. Research investigations showed that various polyphenolic compounds and flavonoids decreased cancer incidence, lowered the risk of developing different cancer types, and reduced cancer relapse [29]. Polyphenols are one of the greatest areas of research for cancer treatment as they confront cancer hallmarks and attack cancer cells efficiently [30]. They possess antioxidant effects through scavenging reactive oxygen species (ROS) and reactive nitrogen species (RNS) as benzene ring-bound hydroxyl groups provide hydrogen atoms or electrons to the free radicals, stabilize them and protect the cellular components [31]. However, in flavonoids, neutralization of the free radicals is performed by a free 3-OH group. Polyphenols also hold the capacity to inhibit the production of ROS and RNS by interfering with some essential enzymes, including nitric oxide synthases, xanthine oxidase, and peroxidase. On the other hand, polyphenols are known to induce cell death by altering the expression of apoptosis-related genes [32]. For instances, curcumin (subclass of polyphenol) induces apoptosis in cancer cell by decreasing ROS, activating mitogen-activated protein kinase (MAPK) signaling pathway, enhancing intracellular Ca influx and activating calcium/calmodulin-dependent protein kinase II (CaMKII) signaling, inducing BCL-2-associated X protein (BAX) and cleaving caspase 3 [33]. Resveratrol and EGCG also possess anticancer properties including impairment of glycolysis, arresting cell cycle of cancer cell and enhancement of apoptosis [34].

#### 2.3.3. Terpenoids

Terpenoids are one of the largest diversified groups of naturally occurring phytoconstituents. They are generally classified as monoterpenoids (C10) (e.g., linalool, geraniol), sesquiterpenoids (C15) (e.g., artemisinin, parthenolide), diterpenoids (C20) (e.g., oridonin, triptolide) and triterpenoids (C30) (e.g., alisol, betulinic acid, oleanolic acid, platycodin D, and ursolic acid) based on the number of carbons formed by isoprene units they contain [35]. Each subclass has its own anticancer activities targeting different cancer developmental stages like initiation, proliferation, angiogenesis and metastasis. Terpenoids influence epigenetic regulations and cellular death pathways by targeting several signaling pathways, including NF-κB, JAK-STAT pathway, Activator Protein-1 and matrix metalloproteinases [36]. Moreover, they activate the p53 gene, modify DNA minor groove structure, interact with DNA polymerase I and II, and inhibit endoplasmic reticulum calcium ATPase pump. They help to ensure cell death by apoptosis mediated by autophagy, which acts as a double-edged sword for cellular survival [37]. Artemisinin triggers G2/M of breast cancer cells and arrests the cell division by enhancing B1, C1 and D cyclins. Another terpenoid, parthenolide, shows anticancer effects against colorectal, pancreatic, breast, prostate, renal and thyroid cancer through the induction of oxidative stress, disruption of mitochondrial function, inhibition of NF-κB and PAMT pathway [38].

### 2.4. Mechanisms of Action of Key Bioactive Natural Compounds in Cancer Therapy

**Figure 1:**
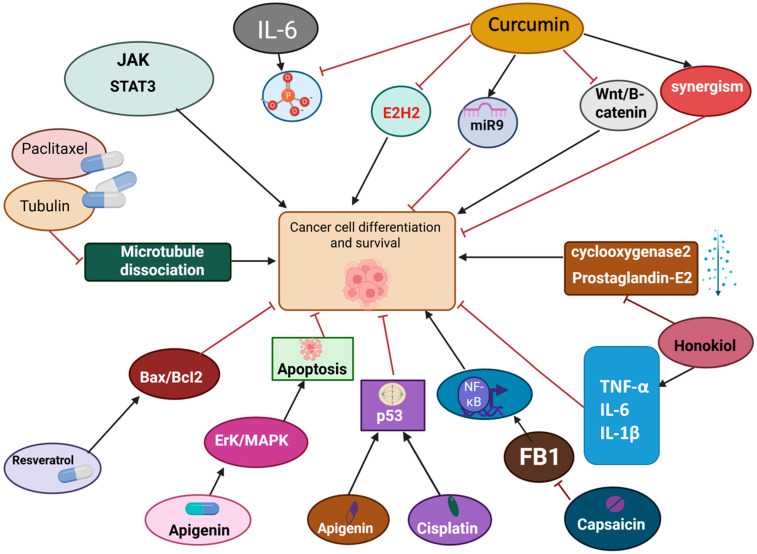
The pathways regulated by natural compounds involved in cancer cell differentiation and survival. Cancer cells demand the hyperactivation of the JAK/STAT pathway, zeste homolog 2 (EZH2), and Wnt/B-catenin pathway. Curcumin suppresses cancer cell progression by not only inhibiting these pathways but also promoting miR-9 and synergism, which ultimately leads to the prevention of cancer cell survival. Other compounds like Resveratrol and apigenin halt cancer progression by upregulating Bax/Bcl2 and ERK/MAPK pathways, respectively. The combination of apigenin and cisplatin has been proven to be effective against cancer cell differentiation since it upregulates p53. On the other hand, FB1 is negatively affected by another bioactive compound, capsaicin, which downregulates NF-κB (cancer cell upregulator). Moreover, honokiol increases the synthesis of TNF-α, IL-6, and IL-1β, while downregulating cyclooxygenase-2 and prostaglandin E2, resulting in a challenging environment for cancer cells. This figure was made by Anup Kumar Bishwas and Created in BioRender. Md. M.N. Azim (2026) https://app.biorender.com/illustrations/693be81010e69a66bcaff066, accessed on 20 January 2026.

Several bioactive natural compounds act as crucial game changers and impressive adjuvants in cancer therapy as they target important signaling pathways and alter the cellular and molecular activity. Since there are different classes of natural compounds, their working mechanisms are distinct from one another. The following section encompasses the mechanism of action of key bioactive natural compounds targeting cell signaling pathways, immunomodulation and immune checkpoints of cancer cells (Figure 1).

Curcumin, regarded as a pleiotropic molecule, is widely popular for its effectiveness in various physiological and pathological processes such as infection, inflammation, skin diseases, hepatic diseases and cancer [39]. Curcumin works against cancer cells by mainly (1) suppressing the JAK2/STAT3 signaling pathway: IL-6 enhances the differentiation and survival of cancer cells through STAT3 phosphorylation. Curcumin successfully stops the phosphorylation of STAT3, hence, blocks the cascade of the JAK/STAT3 pathway. The experiment was conducted in nude mice’s lung cancer of NCI-H460 cell tumorspheres [40]. (2) Exhibiting epigenetic regulatory effect on miRNA and EZH2: miRNA has been a potential target by curcumin for anti-cancer therapy as it is involved in modulating the pathological processes in cancer, and curcumin inhibited zeste homolog 2 (EZH2), which is strongly associated with tumor malignancy. (3) Regulating miR-9 and Wnt/B-catenin pathway: curcumin was found to inhibit squamous cell carcinoma (SCC-9) by upregulating miR-9 (known for reducing cell growth and proliferation, promoting apoptosis in pancreatic cancer cells, and suppressing histone lysine methyltransferase (SET8) expression) and downregulating Wnt/B-catenin expression [41]. (4) Synergism with other anti-cancer agents: The synergistic effect of curcumin with doxorubicin has been proven to be effective against tumor initiation, evasion, and migration by upregulating caspase-9 and Bax while downregulating Bcl-2 expression in a gastric cancer cell line [42]. Another natural anti-cancer compound, paclitaxel, binds to tubulin and guides its assembly into microtubules. The microtubules are now prevented from dissociating by the activity of paclitaxel, which ultimately blocks the progression of the cell cycle and division of cancer cells. Moreover, it has been reported to be involved in various signal transduction pathways such as the toll-like receptor-4 dependent pathway, Janus kinase (JAK) signal transducer and activator of transcription factor pathway, apoptosis by inducing tumor suppressor gene (p53) pathway and in breast cancer, the NF-κB pathway [43].

Many other natural compounds, such as resveratrol, apigenin, capsaicin, honokiol, etc., partially share the pathways mentioned above. But their mechanism of action also significantly differs in the aspect of cell signaling pathways and immunomodulatory actions. For instance, resveratrol is reported to block cell differentiation by inducing mitochondrial-mediated and caspase-independent apoptosis in transgenic adenocarcinoma of mouse prostate cells, where it shows anti-cancer effects by changing the expression of Bax/Bcl-2 and disturbing the mitochondrial membrane potential [44]. Another one, apigenin, was reported to induce apoptosis by the ERK/MAPK pathway. Moreover, the combination treatment with apigenin and cisplatin induced caspase-dependent apoptosis in A549 cells, upregulated the expression of the p53 gene, and enhanced p53 phosphorylation and accumulation in A549 cells [45]. Capsaicin is another promising bioactive natural compound that shows anti-cancer properties by inducing apoptosis via suppression of proto-oncogene, FB1-mediated NF-κB cell signaling pathway in breast cancer cells [46]. In the triple-negative breast cancer cell line, MDA-MB-231, capsaicin has been reported to lower the expression of cyclin-dependent kinase 8 (CDK8), halt cancer cell viability, and trigger cell cycle arrest at the G2/M phase. Another natural compound, honokiol, shows anti-cancer activities by masking the expression of cyclooxygenase-2, prostaglandin E2, propagating cell nuclear antigen, and tumor necrosis factor-alpha, IL-6, and interleukin (IL)-1β in skin cancer [47].

## 3. Nanoparticle Types and TME Modulation in Cancer Therapy

Nanoparticles have emerged as transformative platforms in cancer therapy, offering diverse types from carbon-based to lipid formulations that enable precise drug delivery, imaging, and theranostics while exploiting tumor vulnerabilities. This section examines their classification alongside targeted modulation of the tumor microenvironment (TME), highlighting strategies that enhance penetration, reprogram stroma, and boost antitumor immunity for superior clinical outcomes.

### 3.1. Types of Nanoparticles

Nanoparticles have materialized as highly versatile and promising platforms for cancer therapy, diagnosis, and theranostic applications [48,49]. Carbon-based nanoparticles, including fullerenes, graphene, carbon nanotubes, carbon nanofibers, and carbon black, propose exceptional mechanical strength, electrical conductivity, and light absorption, permitting efficient drug and gene delivery, photothermal therapy, imaging, and tumor scaffolding (Table 2). Metal-based nanoparticles such as aluminum, iron, silver, gold, cobalt, and zinc exploit their reactivity, magnetic properties, and optical responsiveness to achieve targeted drug delivery, hyperthermia, photothermal therapy, and concentrated tumor imaging [50]. Paramagnetic metal oxide nanoparticles, including TiO_2_, Fe_3_O_4_, SiO_2_, ZnO, and Al_2_O_3_, provide stable, magnetic, and highly operable scaffolds suitable for MRI, magnetic targeting, tumor surface coatings, and co-delivery of chemotherapeutics or nucleic acids. Polymer-based nanoparticles, such as dendrimers, polymeric nanoparticles, polymeric micelles, and PEG-PAsp (DIP)-CA micelles, link up biodegradability, surface tunability, and controlled or pH-responsive drug release, promoting tumor-specific accumulation and overcoming multidrug resistance. Lipid-based nanoparticles, including liposomes, solid lipid nanoparticles, and nanostructured lipid carriers, offer biocompatible, high-encapsulation carriers for anticancer drugs, peptides, and proteins, proficiently exploiting the enhanced permeability and retention (EPR) effect for passive tumor targeting [51]. Furthermore, hybrid and inorganic nanoparticles, such as porous silica nanorattles, gold nanoshells, M13 bacteriophage, and optical CNTs, assimilate therapeutic and imaging functionalities to form multifunctional theranostic systems capable of simultaneous tumor surveillance and treatment. Across these diverse platforms, strategic surface alterations, ligand-mediated targeting, and stimuli-responsive release mechanisms collectively enhance tumor specificity, improve therapeutic efficacy, and minimize systemic toxicity, positioning nanoparticles as a cornerstone of next-generation anticancer strategies.

### 3.2. Nanoparticle-Mediated Modulation of the Tumor Microenvironment

Nanoparticle-mediated modulation of the tumor microenvironment (TME) aims to overcome the physical, cellular, and immunological barriers that limit drug delivery, sustaining tumor progression. Since the TME is portrayed by a dense extracellular matrix, aberrant stromal activity, disorganized vasculature, resistant cancer stem cells, and immunosuppressive cell populations, nanoparticles are engineered to remodel these components and restore treatment sensitivity [54]. Enzyme-loaded systems degrade collagen and hyaluronic acid, aiming at enhancing intratumoral penetration, while CAF-targeted platforms reduce stromal resistance. CSC-specific nanocarriers exploit surface markers with a view to eliminating relapse-driving cells, and anti-angiogenic siRNA carriers normalize chaotic vasculature. Nanoparticles that interfere with exosome-mediated signaling disrupt tumor–stroma synergy, whereas immune-modulating formulations activate antitumor immunity and silence MDSCs. Together, these approaches transform the TME into a more permeable, immunoreactive, and therapeutically reactive environment.

#### 3.2.1. Remodeling the Extracellular Matrix (ECM)

**Figure 2:**
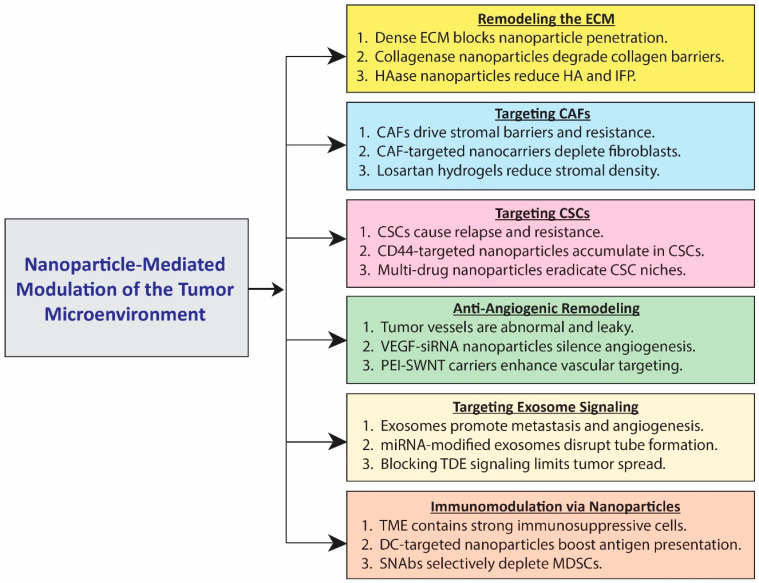
Nanoparticle-mediated designs target multiple tumor microenvironment components. ECM remodeling degrades collagen and hyaluronic acid to enhance drug sensitivity. CAF-targeting downgrades stromal resistance. CSC-specific nanocarriers prohibit therapy-resistant cancer stem cells. Anti-angiogenic nanoparticles coordinate abnormal vasculature. Exosome-targeting systems disintegrate tumor–stroma symbiosis. Immune-modulating nanoparticles activate antitumor immunity and suppress immunosuppressive cells (MDSCs). Collectively, these approaches transform the TME to improve therapeutic reflex.

The extracellular matrix (ECM) forms an intricate, non-cellular, three-dimensional framework, which provides mechanical integrity and biochemical signals promoting cellular functions. Since each tissue type possesses a unique ECM composition, its heterogeneity dictates cell behavior and responsiveness to environmental cues. Within tumors, the ECM does more than occupy intercellular space—it actively contributes to the formation and regulation of the tumor microenvironment (TME) (Figure 2). The ECM comprises collagen, proteoglycans, laminin, and fibronectin, which together generate a dynamic structural network. Initially, the ECM restricts tumor cell migration; however, its abnormal remodeling assists invasion and metastasis [55]. Among ECM constituents, collagen is predominant, which upholds the structural stability of most tissues. In many solid tumors, the ECM is unusually dense and collagen-rich, elevating matrix stiffness and impeding the diffusion of therapeutic molecules and nanocarriers [56]. Reducing collagen content has therefore emerged as an effective strategy to enhance intratumoral nanoparticle penetration. A nano-enzyme delivery platform in which collagenase nanocapsules (Col-nc) were combined with heavy-chain ferritin nanocages encapsulating doxorubicin [57]. In a 4T1 breast cancer model, this construct evolved nanoparticle infiltration by locally degrading collagen, with the nanocapsule shell protecting the enzyme until it reached the tumor site. Hyaluronic acid (HA) is another crucial ECM component—a negatively charged polysaccharide consisting of repeating N-acetylglucosamine and D-glucuronic acid residues—that confers tissue compressive strength and modulates fluid balance. Excessive HA accumulation in tumors increases interstitial fluid pressure and impedes drug infiltration [58]. Enzymatic degradation of HA using hyaluronidases (HAases) has been shown to enhance tissue permeability and nanoparticle diffusion. Developed an enzyme-assisted nanocarrier system (NPs-EPI/HAase), which incorporates HAase to remodel the tumor matrix [59]. HA degradation improved nanoparticle accumulation in HepG2 tumors, resulting in enhanced antitumor efficacy.

#### 3.2.2. Targeting Cancer-Associated Fibroblasts (CAFs)

Cancer-associated fibroblasts (CAFs) are the predominant stromal cell population in tumors that plays a central role in shaping the TME by secreting growth factors (TGF-β, VEGF, PDGF), cytokines, chemokines, and matrix metalloproteinases (MMPs). Through these intermediaries, CAFs promote tumor proliferation, angiogenesis, inflammation, and ECM remodeling [60]. They also inhibit drug delivery and foster therapeutic resistance. Consequently, reprogramming or depleting CAFs has become an important approach to normalize the TME to improve treatment outcomes (Figure 2). Designed a multifunctional stroma-targeted nanocarrier (FH-SSL-Nav), which selectively eliminated CAFs in HepG2 tumor-bearing mice [61]. The system disrupted stromal interactions and partially reversed chemo resistance, thereby indirectly suppressing tumor growth. Similarly, Hu et al. developed a peptide-based hydrogel incorporating losartan, a known CAF-inhibitory agent [62]. Upon intratumoral injection, the hydrogel remained stable for over nine days and released losartan gradually, leading to significant CAF depletion and reduced stromal density in an orthotopic 4T1 breast tumor model.

#### 3.2.3. Targeting Cancer Stem Cells (CSCs)

Cancer stem cells (CSCs) represent a small but highly resilient subpopulation within tumors, capable of self-renewal and differentiation into multiple lineages. These cells are key drivers of tumor initiation, progression, recurrence, and metastasis, contributing substantially to therapy resistance [63]. Conventional chemotherapy and radiotherapy typically eliminate bulk tumor cells while conserving CSCs, allowing residual populations to repopulate the tumor and cause relapse [64]. Therefore, CSC-specific targeting is a vital step toward durable therapeutic responses. Rao et al. fabricated polymeric nanoparticles coated with chitosan, exploiting the strong affinity between chitosan and CD44 receptors copiously expressed on CSCs [65]. This formulation was restrictively accumulated in CD44-positive CSCs within mammary tumor spheroids, achieving superior cytotoxicity. Riboflavin has also exhibited selective uptake in CSCs via ABCG2-mediated transport, which suggests its utility in targeted delivery. Building on this concept, a multifunctional, hypoxia- and pH-responsive nanoparticle system coupled with riboflavin and encapsulating irinotecan, cyclopamine, and erlotinib [66]. Each agent targeted different tumor compartments—CSCs, differentiated cancer cells, and vascular niches—yielding synergistic antitumor activity.

#### 3.2.4. Anti-Angiogenic Remodeling of Tumor Vasculature

Tumor vasculature is typically chaotic, with irregular vessel diameters, poor organization, and leaky lymphatics. These abnormalities impair oxygen and nutrient delivery, which elevates interstitial fluid pressure, contributing to hypoxia, acidosis, and drug resistance [58]. Because tumor expansion beyond 2 mm depends on angiogenesis, disrupting this process depicts a key therapeutic avenue. Compared with traditional anti-vascular agents, nanoparticle-mediated gene silencing offers precise suppression of angiogenic factors. Engineered polyethyleneimine (PEI)-modified single-walled carbon nanotubes (SWNTs) conjugated with candesartan to co-deliver VEGF-siRNA for targeted anti-angiogenic therapy [67]. Candesartan, an angiotensin II type-1 receptor blocker, refined tumor targeting while concurrently downregulating VEGF expression. The PEI-SWNT platform provided efficient intracellular delivery, and in vivo studies in a PANC-1 xenograft model demonstrated marked inhibition of angiogenesis and tumor growth through synergistic mechanisms.

#### 3.2.5. Targeting Exosome-Mediated Communication

Cancer cells exhibit elevated glycolysis rates even under aerobic conditions—a phenomenon linked to their metabolic reprogramming and malignant behavior. Exosomes, a subset of extracellular vehicles (EVs), facilitate intercellular transfer of nucleic acids, proteins, and lipids that profoundly influence tumor progression and remodeling of the TME [68]. Tumor-derived exosomes (TDEs) in particular mediate communication between cancer and stromal cells, intensifying metastasis and immune evasion. Recent evidence reveals that manipulating exosomal microRNAs (miRNAs) can modulate tumor angiogenesis [69]. For instance, in nasopharyngeal carcinoma, lentiviral transfection of cells with miR-9 led to the secretion of miR-9-enriched TDEs that transferred the microRNA to endothelial cells. This transfer inhibited endothelial tube formation and angiogenesis, thereby suppressing tumor progression. Such findings highlight the therapeutic promise of targeting TDE-mediated signaling pathways to interrupt tumor–stroma communication.

#### 3.2.6. Immunoregulatory Modulation via Nanoparticles

Nanoparticles are increasingly being engineered to selectively interact with immune cell populations in the TME, including dendritic cells (DCs), cytotoxic T lymphocytes (CTLs), myeloid-derived suppressor cells (MDSCs), tumor-associated macrophages (TAMs), and regulatory T cells (Tregs). While DCs and CTLs promote antitumor immunity, MDSCs, M2-polarized TAMs, and Tregs exert immunosuppressive effects, which prolong tumor persistence [70]. Targeted delivery to DCs enhances antigen presentation and T-cell activation. Developed biotinylated DEC-205 antibody-modified PLGA nanoparticles encapsulating the MART-1 peptide, an immunogenic melanoma epitope [57]. These nanoparticles induced robust DC maturation and CTL proliferation, which produced a stronger immune response than non-targeted formulations. Conversely, to counteract MDSC-mediated suppression, Liu et al. created synthetic nanoparticle antibodies (SNAbs)—Janus-type multivalent nanostructures coupled with G3 or G3* peptides that bind the MDSC-specific S100A8/A9 complex [71]. In 4T1 murine breast cancer models, SNAbs efficiently depleted MDSCs, restored immune balance, and significantly enhanced antitumor activity.

### 3.3. Current Applications in Nanoparticle-Mediated Cancer Therapy

**Figure 3:**
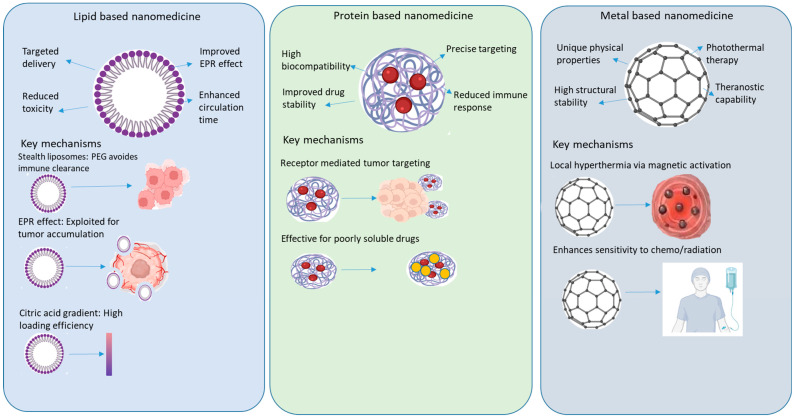
Current landscape of clinically approved nanoparticle-mediated cancer therapies. The schematic illustrates the three major classes of FDA/EMA-approved nanopharmaceuticals: lipid based, protein-based, and metallic systems. (**Left**) Lipid-based nanomedicines (e.g., Doxil™, Vyxeos™) typically range from 80 to 120 nm and utilize PEGylation (“stealth” coating) to prolong circulation and exploit the enhanced permeability and retention (EPR) effect for passive tumor accumulation. (**Center**) Protein-based nanocarriers (e.g., Abraxane™, Kadcyla™) leverage receptor mediated endocytosis (targeting gp60, SPARC, or HER2) to deliver cytotoxic payloads like paclitaxel or antibodies directly to cancer cells. (**Right**) Metallic/Iron-oxide nanomedicines (e.g., NanoTherm™) function primarily through physical mechanisms, such as magnetic hyperthermia induced by an alternating magnetic field, to sensitize or destroy tumor tissue. Abbreviations: EPR, enhanced permeability and retention; PEG, polyethylene glycol; HER2, human epidermal growth factor receptor 2.

Current cancer nanopharmaceuticals include lipid, protein, and metallic-based nanoformulations, which offer targeted delivery, reduced toxicity, and improved efficacy (Figure 3). Innovations in this field mark significant progress and patient outcomes, as shown in Table 3.

## 4. Multidrug Resistance and How Nanoparticle Overcome Drug Resistance

**Figure 4:**
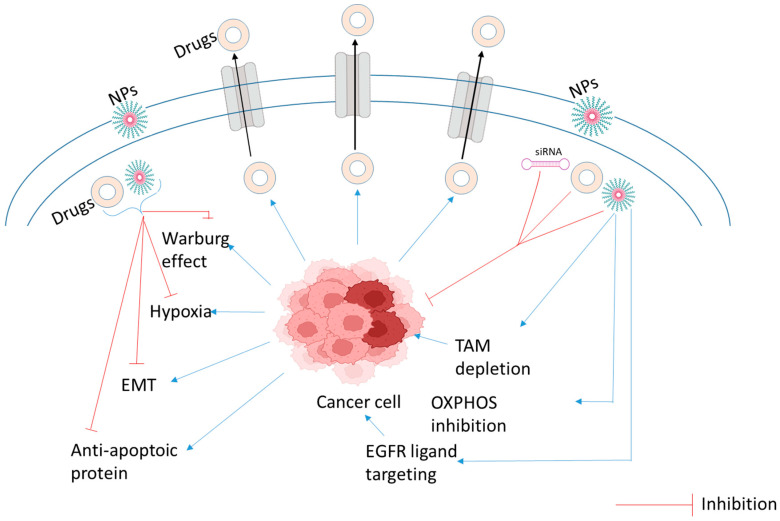
Mechanisms of multidrug resistance (MDR) in cancer and nanoparticle-mediated strategies to overcome them. The key cellular and microenvironmental factors contributing to MDR include the overexpression of drug efflux pumps, activation of anti-apoptotic pathways, hypoxic or acidic TME, metabolic rewiring, and the persistence of cancer stem cells (CSCs) via epithelial–mesenchymal transition (EMT). Nanoparticle strategies designed to circumvent these barriers include: (1) bypassing efflux pumps via endocytosis; (2) co-delivery of chemotherapeutics with siRNA or inhibitors; (3) targeting TME components like tumor-associated macrophages (TAMs); and (4) specific inhibition of metabolic pathways (e.g., OXPHOS) and CSC markers.

Cancer cells develop multidrug resistance (MDR) by learning to tolerate several chemotherapy drugs, lessening treatment effectiveness considerably and increasing the risk of death. The tumor microenvironment, a complex mixture of cancer cells and surroundings, contributes to developing this resistance and cancer metastasis [75]. Cancer cells employ different strategies of resistance from within the cell itself or the surrounding tissue. Drug resistance may be developed by affecting how the body metabolizes the drug or how the drug interacts with the cancer cell (Figure 4). Some strategies involve limiting drug entry, eliminating drugs, repairing DNA damage, changing drug targets, and avoiding cell death. For example, cancer cells can enhance the number of drug pumps to expel chemotherapy drugs, thereby preventing effective drug concentrations from building up inside. Researchers are working to overcome MDR with various strategies, including blocking these drug pumps, inhibiting cancer stem cells, disrupting the cancer’s metabolism, improving the immune system’s ability to destroy the tumor, blocking the cancer cells from becoming invasive, and restoring normal cell death [76]. Even though most current cancer therapies are highly targeted, resistance to drugs remains a significant challenge to long-term success, primarily because cancer cells can drug-proof themselves by ejecting drugs out of their cells through ABC transporters. Another challenge with resistance is from within the tumor, such as cell death pathway problems, fluid pressure, acidity, and oxygen shortage. The application of nanotechnology-based drug delivery offers an answer to bypassing (Figure 4) this by enhancing chemotherapy and facilitating combination therapies [77]. Using nanoplatforms, researchers can deliver MDR inhibitors to resensitize drug-resistant cells. The systems enable the simultaneous delivery of multiple drugs and, therefore, a more potent approach against drug resistance.

### 4.1. Nanocarrier-Based Drug Delivery Systems in Cancer Therapy

**Figure 5:**
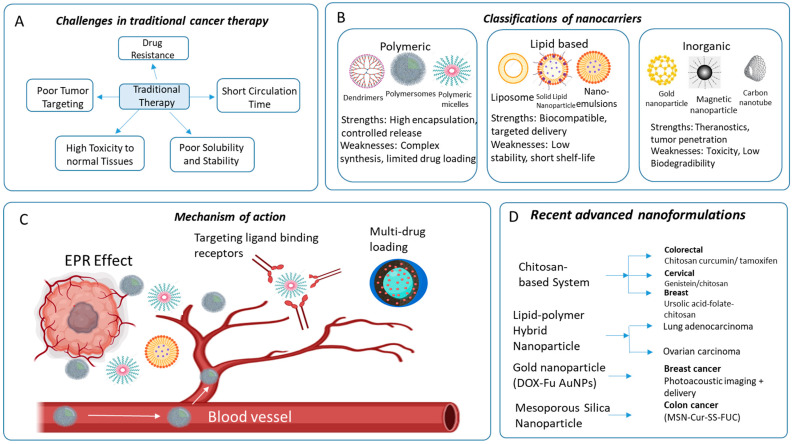
Comprehensive overview of nanocarrier-based drug delivery systems in cancer therapy. (**A**) Schematic illustration of the limitations of traditional chemotherapy (e.g., poor solubility, systemic toxicity, drug resistance) versus the solutions offered by nanotechnology. (**B**) Classification of major nanocarrier types into three categories: Polymeric (dendrimers, micelles), Lipid-based (liposomes, solid lipid nanoparticles), and Inorganic (gold NPs, magnetic nanoparticle, carbon nanotube). (**C**) Mechanisms of tumor accumulation, highlighting passive targeting via the enhanced permeability and retention (EPR) effect and active targeting via ligand-receptor binding. (**D**) Recent advancements in nanoformulations, showcasing specific materials (e.g., chitosan, albumin, mesoporous silica) applied across various cancer types (breast, colon, lung). This figure was made by Anup Kumar Bishwas and (Created in BioRender. Md. M.N. Azim (2026) https://app.biorender.com/illustrations/696f6ecb43c04effdc48382d, accessed on 20 January 2026).

The effectiveness of cancer treatment is limited by several factors, including drug resistance, ineffective tumor targeting, reduced circulation time of drugs, and cytotoxic side effects. A promising way to address these issues is through nanocarrier-based drug delivery systems. These systems include various types of nanocarriers, such as liposomes, dendrimers, micelles, solid-lipid nanoparticles, and carbon nanotubes, which hold potential for overcoming current limitations in cancer therapy [78]. Nanocarriers provide a new method of drug delivery that is more targeted and effective. Researchers are exploring nanocarriers, especially those between 10 and 100 nm, as promising tools in cancer treatment (Figure 5). These nanoscale carriers surpass traditional approaches by improving drug solubility, distribution, and targeting, while also decreasing toxicity [79]. Due to their small size, they can cross physiological barriers, leading to higher drug concentrations in cancer cells and fewer side effects in normal tissues. Using multiple drugs within nanocarriers can further enhance treatment efficacy. By adjusting the size and shape of nanocarriers, scientists can precisely control how drugs behave in the body. Anticancer drugs are delivered through various nanocarriers, including liposomes, dendrimers, carbon nanotubes, metallic nanoparticles, solid lipid nanoparticles, nanostructured lipid carriers, polymeric nanoparticles, and polymeric micelles [80]. Polymeric nanocarriers include polymersomes, dendrimers, micelles, nanogels, and nanocapsules, while lipid-based nanocarriers comprise liposomes, solid lipid nanocarriers, phospholipid micelles, and nanoemulsions. Inorganic nanocarriers such as magnetic, silicon, gold nanoparticles, carbon nanotubes, and quantum dots also play important roles in drug delivery. Solid-lipid nanoparticles offer increased bioavailability, controlled release, and targeted delivery of water and fat-soluble medications [81]. They have drug stability with low toxicity, but agglomeration is an issue. These nanoparticles optimize the therapeutic potential of anticancer medications. Carbon nanotubes offer biocompatible medication delivery with the potential to cross biological barriers and versatile modifications. Dendrimers are excellent for drug delivery to tumors due to their high permeability and retention. Their superiority in drug encapsulation and identical size make them ideal vehicles [82]. Micelles possess excellent advantages in drug delivery, including high drug loading and stability [83]. Their hydrophilic nature promotes solubility, and they are typically low in toxicity. They are, however, hampered by limited drug incorporation, failure to reach target tissues, and potential liver toxicity from slow metabolism. Their small size and complex polymer synthesis also deter standardization and mass production. Metal and magnetic nanoparticles, being small in size (10–100 nm), can penetrate blood vessels in most tissues and enhance drug accumulation in tumors. This allows for the potential of improved diagnosis and treatment. Their use is, however, limited by issues that range from low stability to limited compatibility with biological systems and the potential for high toxicity.

Lipid–polymer hybrid nanoparticles are being researched for extended cancer therapy, including against lung adenocarcinoma, ovarian carcinoma, liver cancer, and embryonal carcinoma [84]. Nanomaterials based on chitosan have also been explored for drug delivery in various cancers, such as genistein-loaded nanomaterials for cervical cancer, ursolic acid-loaded folate-chitosan nanomaterials for breast cancer, and isolongifolene-loaded chitosan nanomaterials for the treatment of lung cancer [85]. Isolated from Fucus vesiculosus, nanovesicles of fucoidan also possess potential for chemotherapy against lung carcinoma and melanoma cells. Furthermore, lactoferrin nanoparticles have been utilized as a novel drug delivery platform for pancreatic cancer. Doxorubicin-conjugated gold nanoparticles (DOX-Fu AuNPs) integrate photoacoustic imaging and drug delivery functions for breast cancer therapy. Alginate-based drug delivery systems have also been created for targeted cancer therapy [86]. Alginate of *Laminaria hyperborea* has been blended with poly(D, L-lactide-co-glycolide) (PLGA) to form microspheres for chemotherapy and targeted drug delivery in the therapy of colon cancer. Oral administration of curcumin diglutaric acid nanoparticles comprising chitosan/alginate has been developed for the therapy of human epithelial colorectal adenocarcinoma. Pectin-based systems of nanoparticles are under investigation for their application in cancer therapy [87]. For gastric cancer, the delivery of pectin is enhanced by N-acetyl-D-glucosamine. Hyaluronic acid-coated iron oxide nanoparticles are also engineered to target doxorubicin delivery for improved breast cancer therapy. Dextran-curcumin nanoparticles have also been developed as a drug delivery system for methotrexate in the treatment of breast cancer [88]. 1,3-β-glucan derived from Lentinula edodes was also used to encapsulate curcumin to enhance drug targeting and hydrophilicity towards the treatment of hepatocellular carcinoma. Thunnus obesus-derived collagen (fish skin, bones, and scales) was used to develop paclitaxel-encapsulated collagen nanoparticles containing sustained release of the drug for the treatment of breast cancer. In addition, doxorubicin encapsulated within collagen peptide-chitosan nanoparticles presented efficient drug delivery with good biocompatibility in the treatment of cervical cancer. Albumin, which may be derived from bovine serum or egg albumin, has been utilized as part of nanoparticle-based drug delivery systems in anticancer therapy.

### 4.2. Anticancer Properties and Drug Delivery Potential of Marine and Plant-Derived Biopolymers

**Figure 6:**
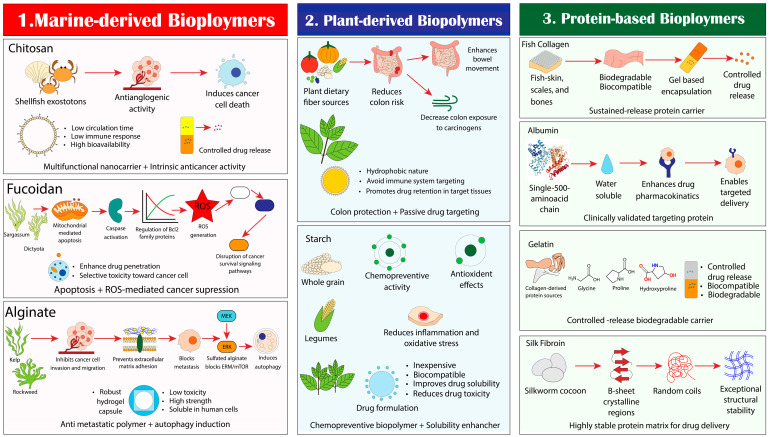
Therapeutic potential and drug delivery applications of natural biopolymers in oncology. Biopolymers derived from marine, plant, and protein sources contribute to a major role in cancer therapy and drug delivery because of their biocompatibility, biodegradability, and multifunctional biological activities. Chitosan, acquired from shellfish exoskeletons, shows anti-angiogenic activity, induces cancer cell death, and facilitates controlled drug release with minimal immune response. Fucoidan, sourced from brown seaweeds, elevates mitochondrial-mediated apoptosis through caspase activation and reactive oxygen species generation, resulting in selective toxicity toward cancer cells. By forming low-toxicity hydrogel systems, Alginate (extracted from seaweed) inhibits cancer cell invasion and metastasis, blocks ERK/mTOR signaling pathways, and induces autophagy. Although starch from whole grains and legumes exhibits antioxidant and anti-inflammatory effects, plant-derived biopolymers assist in chemoprevention and passive drug targeting. However, dietary fibers lessen colon cancer risk by enhancing bowel movement and limiting exposure to carcinogens. Protein-based biopolymers, including fish collagen, albumin, gelatin, and silk fibroin, are widely used for controlled and sustained drug delivery. Fish collagen provides biodegradable and biocompatible gel-based encapsulation, while albumin, which is a clinically validated carrier, improves pharmacokinetics and ensures targeted delivery. Gelatin offers controlled release and biodegradability, whereas silk fibroin constructs a highly stable protein matrix with exceptional structural integrity, making it suitable for advanced drug delivery applications.

Chitosan, a polymer derived from shellfish exoskeletons, has anti-angiogenic activity and induces cancer cell death. It is one of the most widely used nanocarriers because it offers prolonged circulation, low immune response, good bioavailability, and excellent biocompatibility. Chitosan not only stabilizes drugs in serum but also reduces side effects and enables controlled drug delivery. Fucoidan, a sulfated polysaccharide from *Sargassum wightii*, *Dictyota dichotoma*, and *Turbinaria ornata* seaweeds, inhibits cancer by inducing mitochondrial-mediated apoptosis and disrupting cancer cell survival signal pathways (Figure 6). It impairs the growth of cancer cells at all cell cycle stages by modulating BCL-2 family proteins and activating caspases. Fucoidan also stimulates reactive oxygen species (ROS) within cells, enhances drug penetration, and selectively destroys cancer cells. Alginate, a seaweed-derived polymer from *Ascophyllum nodosum* and *Laminaria hyperborea*, prevents cancer metastasis by blocking cell invasion and migration (Figure 6). It impedes cancer cell adhesion to the extracellular matrix, thereby inhibiting spread. Sulfated alginate promotes autophagy and exhibits anticancer effects through inhibition of the MEK1/ERK/mTOR pathway. Its high strength, low toxicity, and excellent biocompatibility make alginate a suitable material for drug delivery. Since alginate is soluble in human cells lacking digestive enzymes, it is suitable for delivery. Dietary fiber, cellulose from plants, lowers the risk of colon cancer by promoting normal bowel movements and minimizing the colon’s exposure to carcinogenic chemicals. Additionally, starch is inexpensive, biocompatible, and enhances drug solubility while reducing toxicity.

Fish collagen, a triple-helix protein located in scales, skin, and bones, possesses several advantages [89]. It is innocuous to use biologically, biodegradable, and compatible for encapsulation in gel-based form. The collagen also allows for the controlled release of drugs, improving bioavailability. Albumin, a water-soluble protein with a single 500-amino acid chain that is folded into three similar domains, can be used for targeted drug delivery. It is biocompatible and biodegradable, and enhances the pharmacokinetics of drugs. Gelatin, a protein derived from triple-helical collagen, is mostly composed of glycine, proline, and hydroxyproline. It offers controlled drug release, enhancing drug delivery, and is biocompatible and biodegradable. Silk fibroin, derived from Bombyx mori silkworm cocoons, has a very stable secondary structure. The stability is due to its structure being made up of β-sheet crystals alternating with random coil and α-helical parts.

## 5. Recent Advances in Preclinical and Clinical Studies with Nanoparticles in Tumor Therapeutics

Preclinical trials provide crucial information on efficacy, toxicity, and safety to make clinical trial decisions. More recently, intensive in vivo studies have yielded conclusive proof for the potential anti-cancer activity of various bioactive phytochemicals against different types of malignancies, again supporting their integration into advanced nanoparticle delivery systems [90]. Within the context of lung and respiratory malignancies, 6-shogaol, of which the molecular target is the ginger root compound, significantly inhibited the proliferation of various malignancy cells through suppression of cell proliferation and induction of apoptosis via the down-regulation of Akt signaling [91]. Glycyrrhizin significantly suppressed lung malignancy progression through down-regulation of the Thromboxane A2 pathway and reduction in proliferating cell nuclear antigen expression [92], while Emotionol from *Rheum palmatum* L. significantly induced apoptosis in lung cancer cells via endoplasmic reticulum stress and TRIB3/NF-κB pathway signaling. More recently, baicalein and baicalin from *Scutellaria baicalensis* Georgi significantly suppressed mammalian colon malignancy progression, whereby baicalein suppressed malignancy through the down-regulation of p-38, ERK, and MAPK pathways [93]. On the other hand, baicalin significantly suppressed malignancy progression through down-regulation of c-Myc and oncogenic microRNAs. While genistein significantly suppressed the development of aberrant crypts in experimentally induced colon malignancy models in rats via down-regulation of the Wnt pathway and subsequent β-catenin nuclear expression, Hispidulin from *Saussurea involucrata* significantly suppressed malignancy progression in hepatomas and lung malignancy metastasis, whereby it activated PPARγ and AMP-kinase pathways [94]. Finally, decursinol from Decursio holodictyoides significantly suppressed malignancy growth and hematogenous spread in malignancy models, while alpinumisoflavone from *Alpinia ogonosperma* significantly suppressed malignancy growth in renal malignancies, whereby it up-regulated miR-101 and suppressed Akt signaling. Lastly, dicumarol significantly suppressed malignancy growth in murine ovarian malignancy cells [95]. The above findings collectively emphasize and validate, through several preclinical studies, the overall anti-cancer activity of phytochemicals and offer a sound biological rationale for effectively integrating phytochemicals into nanoparticle-mediated delivery systems.

Although most phytochemicals with anticancer properties are still in the early stages, there has been a steady increase in the number entering clinical trials, mainly as phase II studies focusing on their chemopreventive properties, safety, and role as adjuncts in mainstream cancer therapy. For prostate cancer, the phytochemical sulforaphane has been the most extensively studied. A phase II trial with 78 patients showing rising prostate-specific antigen levels after prostatectomy found that 60 mg of sulforaphane taken orally safely extended the PSA doubling time This aligns with another trial in patients with biochemically recurrent prostate cancer, which used 200 µmol/day of broccoli sprouts and also showed a significant extension of PSA doubling time [96]. In colorectal and gastrointestinal cancers, the phytochemical berberine is now in late-phase clinical trials. This is based on a phase III trial where patients took 300 mg of berberine hydrochloride twice daily to prevent new adenomas in those with a history of colon adenocarcinoma [97]. Besides gastrointestinal cancers, EGCG-based strategies are actively being investigated to prevent and treat breast, ovarian, and skin cancers [98]. Additionally, the therapeutic potential of sinecatechins, a green tea extract, is being studied in mid-stage trials for superficial skin malignancies. Quercetin, another phytochemical, is currently under investigation in early-phase clinical studies for its potential to prevent chemotherapy-induced mucositis [99]. Overall, these trials highlight the translational promise of phytochemicals and emphasize the need for larger studies to fully assess their potential.

## 6. Intellectual Property Landscape: Nanomedicine and Bioactive Natural Products

The intersection of nanotechnology and natural product-based oncology has yielded a rapidly widening patent landscape (Table 4), propelling further explorations designed to surmount the poor bioavailability, systemic toxicity, and low target specificity often associated with phytochemical usage. More recent patents reflect a strong focus on the development of novel nanocarriers for the stabilization and selective delivery of such potent agents to tumor sites.

## 7. Challenges and Emerging Trends

### 7.1. Toxicity

Nanoparticles (NPs) are widely used in nanomedicine and as drug nanocarriers because of their small size and unique properties. However, their size, shape, surface functional groups, and dose-related characteristics can also lead to toxicity in normal, healthy human cells, tissues, and organs (Table 5). Many studies show that chemically made nanoparticles tend to be more toxic to human cells because they contain synthetic chemicals as surface functional and capping agents, unlike biosynthesized nanoparticles that have biocompatible surface groups [101]. On the other hand, some biosynthesized nanoparticles can also be toxic when interacting with cells, either by breaking down into simpler forms or by building up in the body. when the size of a nanoparticle decreases from 30 to 3 nm, the proportion of surface molecules expressed rises from 10% to 50%. The cytotoxicity of nanomaterials results from interactions between their surfaces and cellular components. Therefore, even if nanoparticles share the same chemical composition, their cytotoxicity can vary significantly depending on surface area and particle size. In other words, nanoparticles are more toxic than larger particles with similar compositions. Studies have examined the cardiovascular toxicity of amorphous silica nanoparticles of different sizes (90, 60, and 30 nm) and 600 nm fine silica nanoparticles after intratracheal instillation in rats [102]. The subcellular localization, toxicological effects, and tissue distribution of three distinct sizes of gold nanoparticles were assessed. Particles measuring 10, 30, and 60 nm were used, and their distribution was evaluated after intraperitoneal injection in rats. The 10 nm and 30 nm gold nanoparticles crossed the nuclear membrane, leading to DNA breaks. These smaller particles also accumulated more in the liver, kidney, and intestine compared to 60 nm gold nanoparticles, which predominantly concentrated in the spleen. Consequently, larger gold nanoparticles tend to accumulate more in the spleen. In summary, nanoparticles have greater surface areas and higher particle counts per unit mass compared to larger particles. The engineered nanoparticles exhibit heightened surface reactivity and a substantial surface area, which can lead to increased levels of reactive oxygen species, ultimately causing cytotoxicity and genotoxicity [103]. The morphology of nanoparticles is a key factor that determines their biological reactivity and toxicity. Common geometries of nanoparticles include spheres, cylinders, cubes, sheets, and rods. The shape of a nanoparticle significantly influences its cellular absorption. Silver nanoplates were found to be more harmful than silver nanospheres in zebrafish (Danio rerio) embryos [104]. Spherical nanoparticles are taken up by cells in greater quantities than other shapes. Gold nanorods result in less accumulation of autophagosomes compared to gold nanospheres. The cytotoxic effects of gold nanoparticles in star, rod, and spherical forms were studied on human fetal osteoblasts, osteosarcoma, and pancreatic duct cell lines using the MTT assay [105]. The star-shaped gold nanoparticles show the highest cytotoxicity toward human cells. Nanoparticle surface coatings are used to modify their properties. The purpose of the surface coating may be to maintain its stability, solubility, or functionality. The coating can convert toxic particles into non-toxic ones, while less harmful particles might become more toxic due to increased bioavailability [106]. An in vitro assessment was conducted on the toxicity of silica-coated iron oxide nanoparticles (Fe_3_O_4_/SiO_2_ NP) on HeLa and A549 cells. Reports indicate that surface passivation of nanoparticles reduces disruption of iron balance and oxidative stress. This results in decreased overall toxicity during cell uptake compared to non-passivated nanoparticles [107]. One study showed that iron oxide nanoparticles coated with polyethyleneimine (PEI) had significantly greater uptake than PEGylated iron oxide nanoparticles in both cancer cells and macrophages, leading to increased cytotoxicity. The dissolution ability of nanoparticles is a key property that influences safety, uptake, and toxic mechanisms. Two identical NPs with similar composition and size may behave very differently in dissolution depending on surface modifications.

### 7.2. Scalability

The main challenge in successfully scaling up and producing nanomedicine is maintaining consistent stability of physicochemical properties across different batches. Most nanoparticles are complex, three-dimensional entities with specific arrangements of multiple components, making the chemistry, manufacturing, and control processes highly demanding. Traditional pharmaceutical manufacturing has limited experience in creating nanoscale, multicomponent systems. All of these factors contribute to the challenges in scaling up and manufacturing nanomedicine [108]. The nanomedical formulation process often involves high-speed homogenization, sonication, milling, emulsification, crosslinking, solvent evaporation, centrifugation, filtration, and lyophilization. It is crucial to evaluate suitable methods and parameters for scaling up during laboratory or small-scale phases, as these critical conditions will influence later development stages [109]. Parameters may include the ratio of nanomaterials, drugs, and targeting agents, choice of organic solvents and stabilizers, oil-to-water phase ratio, temperature, pressure, and pH. Using incorrect conditions could alter the chemical structure of therapeutic agents, affect auxiliary components, or introduce unexpected impurities. The high costs of raw materials and the complexity of multistep production pose significant barriers to scaling and manufacturing nanomedicine, making the output of nano-therapeutics very costly. This can hinder pharmaceutical companies from scaling up the production of nanocarriers.

**Table 5:** Ways to overcome key disadvantages of modern nanocarriers [110,111].

Nanocarrier Challenge	Typical Disadvantages in Current Systems	Possible Ways to Overcome These Disadvantages
Toxicity of inorganic/metal NPs (Au, silica, SPIONs, etc.)	Dose-, size- and shape-dependent cytotoxicity and genotoxicity; ROS generation; off-target accumulation in liver, spleen, kidney and even nucleus.	Biocompatible surface coatings (silica, dextran, PEG, polysaccharides) to reduce surface reactivity and oxidative stress; careful size/shape optimization to favor safe clearance and avoid nuclear entry; use of biosynthesized or biopolymer-coated NPs to lower intrinsic toxicity
Instability, aggregation and rapid clearance	Agglomeration of SLNs and metallic NPs; opsonization, RES uptake and short circulation, reducing tumor accumulation.	PEGylation (“stealth” coating) and cholesterol optimization; use of stabilizing surfactants/polymers; lipid–polymer hybrid NPs to combine structural robustness with biocompatibility; process optimization (homogenization, sonication, lyophilization conditions) to maintain colloidal stability at scale.
Limited tumor penetration and TME barriers	Dense collagen/HA-rich ECM, high interstitial pressure, CAFs, CSCs, chaotic vasculature and immunosuppressive cells restricting nanocarrier delivery.	ECM-remodeling nanocarriers (collagenase or hyaluronidase-loaded systems); CAF-targeted liposomes/hydrogels (e.g., losartan-releasing); CSC-directed NPs (CD44/riboflavin-targeted); anti-angiogenic siRNA delivery; exosome-, TAM- and MDSC-targeting platforms to normalize TME and improve penetration.
Multidrug resistance (MDR)	Efflux pumps (ABC transporters), enhanced DNA repair, anti-apoptotic signaling, EMT, CSC persistence, metabolic rewiring (e.g., OXPHOS).	Endocytic uptake of drugs via NPs to bypass efflux; co-delivery of chemotherapeutics with siRNA or small-molecule inhibitors against pumps, survival pathways or metabolic targets; TME- and CSC-targeted nanocarriers; use of chemosensitizers within the same platform.
Limited loading, targeting and standardization of polymeric/micellar systems	Limited drug incorporation, failure to reach target tissues, potential liver toxicity, complex polymer synthesis hampering scale-up.	Rational polymer design (biodegradable, low-toxicity, simpler architectures); stimuli-responsive (pH, redox, enzyme) micelles for on-site release; combining micelles with liposomes or hybrids; early CMC and scale-up optimization to ensure reproducible critical quality attributes.
Scalability and cost of manufacturing	Multistep processes (homogenization, milling, emulsification, solvent removal, lyophilization); batch-to-batch variability; high material and process costs.	Process-intensified, scalable unit operations (continuous emulsification, microfluidics); predefined design-space for key parameters (ratios, solvents, pH, temperature, pressure); selection of cheaper, robust excipients and simpler nanostructures; early alignment with GMP and quality-by-design principles.
Regulatory uncertainty and variable biodistribution	Lack of specific guidelines; complex behavior in vivo (protein corona, altered biodistribution, organ accumulation).	Thorough physicochemical characterization (size, shape, charge, dissolution, coating); standardized in vitro and in vivo toxicology panels; development of “smart” and AI-designed nanocarriers with well-defined, measurable critical quality attributes to support clearer regulatory assessment.

### 7.3. Regulatory Hurdles

Although the FDA and the European Medicines Agency (EMA) have approved several nanomedicine products for cancer treatment, specific guidelines for drug products containing soft materials have not yet been established by these agencies or other regulatory authorities [112]. The lack of guidance on evaluating nanomedicine means that regulatory decisions rely mainly on individual assessments of benefits and risks. This process can be lengthy and requires expertise in innovative technologies, which may cause delays. Regulatory issues are essential for the progress of new technologies that define and monitor the quality of nanomedicine products, aside from clinical trials and approval procedures [113]. There is an urgent need for comprehensive guidelines on characterizing and ensuring the quality of nanomedicine products. Furthermore, biological challenges exist, such as limited routes of administration and altered biodistribution. After administration, nanocarriers or their degradable soft components may come into contact with biological fluids, like blood serum, or biomolecules, such as proteins.

### 7.4. Emerging Trends

Smart nanoparticles (NPs) have become a promising alternative to traditional nanoparticles for cancer treatment. Unlike conventional ones, they can be triggered by specific stimuli and target particular sites with precise drug delivery. After modification or stimulation by relevant factors, these smart nanoparticles efficiently gather at the target site and release their payloads, creating an intelligent treatment system. They can carry and release anticancer drugs to specific areas for accurate cancer therapy. However, the fate of these drug-carrying nanocarriers remains a concern. Conventional nanoparticles can accumulate in the lungs, spleen, kidneys, liver, and heart, depending on their chemical makeup, size, shape, surface area, charge, and whether they have a shell. Recently, AI-powered nanoparticles have emerged as a promising approach for cancer therapy, offering potential improvements in personalized medicine [114]. Personalized medicine can be defined as a customized management strategy aimed at administering the appropriate drug at the correct dosage to the suitable patient. Personalized medicine encompasses proteomic, genomic, and epigenetic analyses, alongside particular patient health conditions and environmental factors [115]. Nanomedicine has been associated with the prevention, monitoring, diagnosis, and treatment of diseases, and numerous innovations in this field are utilized daily in contemporary clinical practice. The convergence of nanotechnology and personalized medicine occurs at various junctures. Initially, the diagnostic domain presents significant opportunities for nanotechnology in assessing specific drug targets, conducting pharmacogenetic testing, and facilitating both in vitro and in vivo evaluations. Secondly, the therapeutic domain allows nanomedicine to customize the drug for a particular target associated with a specific disease in an individual patient.

Compared to traditional drug delivery systems, nanodrugs offer many pharmacokinetic benefits. These include improved solubility and absorption, potential for controlled release, increased drug stability and metabolism, reduced side effects, longer blood circulation, and better effectiveness in targeted delivery [116]. Aptamers are short single-stranded RNA or DNA sequences created through in vitro selection of randomized oligonucleotides using Systematic Evolution of Ligands by Exponential enrichment (SELEX). They specifically bind to molecules such as peptides or proteins. Aptamers have greater conformational stability than antibodies and can reversibly refold to their original shape. Furthermore, RNA-based aptamers like A15 have been developed to improve blood–brain barrier penetration, and a transferrin-receptor aptamer liposomal delivery system showed better uptake in the rodent brain compared to control liposomes. The use of siRNAs represents a practical gene-silencing method useful in antiviral and antitumor therapies. RNA interference-based spherical nucleic acids (SNAs), composed of a gold nanoparticle attached to siRNA oligonucleotides targeting the highly expressed GBM oncogene Bcl2Like12 (Bcl2L12). The siRNA effectively silenced Bcl2L12, activated caspase-3, and increased wild-type p53 protein levels. Gapmers are short chimeric antisense oligonucleotides with a central DNA core flanked by 20-O-methylated RNA-like sequences. When in the presence of glutathione, the nanoparticle structure undergoes degradation, releasing the p53-targeting gapmer. This process significantly reduced cell proliferation in PANC-1 and MDA-MB-231 cancer cell lines, which have p53 mutations, while not affecting MCF-7 cancer cells.

## 8. Future Research Directions

Future research on bioactive natural products and nanoparticles for cancer drug delivery should focus on integrating mechanistic understanding, innovative formulations, and pathways for clinical translation. Discovery based on mechanisms—supported by structure–activity relationship studies and semi-synthetic modifications—will be key to improving the potency, selectivity, and pharmacokinetics of potential compounds. Advances in nanotechnology should be leveraged to develop carriers that enhance solubility, stability, and tumor-specific accumulation, while enabling controlled and stimulus-responsive drug release. Combining natural compounds with conventional chemotherapies, targeted therapies, or immunotherapies offers promise for overcoming resistance and reducing toxicity, and should be tested in advanced preclinical models like patient-derived xenografts, tumor organoids, and syngeneic systems that better reflect tumor diversity and immune responses. Designing nanoparticles capable of penetrating dense tumor stroma, modifying the tumor microenvironment, and bypassing mechanisms of multidrug resistance will boost treatment effectiveness. Efforts should also be directed toward identifying predictive biomarkers to assist in patient selection, optimize dosing, and guide clinical trial designs. Furthermore, sustainable sourcing or synthetic biology approaches for producing scarce natural scaffolds, along with scalable and GMP-compliant manufacturing processes, will be essential for transitioning these nanomedicines into clinical use. Addressing these scientific, technological, and regulatory challenges will help natural product-based nanomedicines move from promising laboratory results to safe, effective, and precisely targeted cancer therapies.

## 9. Conclusions

The combination of bioactive natural compounds with nanotechnology has proven to be a revolutionary approach in developing anticancer therapies. As discussed in this review, natural compounds like alkaloids, polyphenols, and terpenoids exhibit strong multi-target anticancer activity, ranging from inducing apoptosis to inhibiting angiogenesis. However, their use in cancer treatment has been limited in the past due to issues with poor solubility, stability, and bioavailability. The application of nanoparticles as delivery systems has emerged as the ideal solution to these biopharmaceutical challenges associated with natural compounds. By utilizing various nanocarriers such as lipid-based, polymeric, and metallic nanocarriers, the EPR effect can be exploited to maximize drug accumulation in tumors while minimizing systemic toxicity. The major obstacle, multidrug resistance, has been addressed through the use of nanoparticulates and chemosensitizers. The future of cancer treatment will focus on developing “smart” nanoparticles and artificial intelligence for precision medicine. Precision medicine can provide targeted treatments based on an individual’s genome and proteome, while nanotechnology can be used effectively for delivering precise doses of medications. This approach promises improved efficiency and fewer side effects. Bridging the gap between laboratory success and real-world application requires interdisciplinary collaboration among innovators and developers of bio/nano-fusions, aiming to make life-saving treatments accessible and practical for all cancer patients worldwide. This review uniquely integrates nanocarrier limitations with bioactive compound synergies in the tumor microenvironment, offering a novel framework for TME-modulating designs not previously synthesized or “By bridging genetic engineering strategies with clinical translation gaps, this work pioneers actionable recommendations for next-generation antioxidant-enriched therapies.”

## Figures and Tables

**Table 1:** Bioactive natural products with their natural origin of each compound and activities.

Bioactive Compounds	Natural Origin of Each Compound	Activity	Reference
Flavonoids	Plant secondary metabolites widely distributed in higher plants (e.g., *Morus alba*)	Cell cycle arrest	[17]
Alkaloids	Plant secondary metabolites with nitrogen-containing heterocycles (e.g., *Annona muricata*)	Apoptosis by arresting the G1 phase, accumulation of ROS and mitochondrial membrane potential disruption	[18]
Quercetin	Polyphenolic flavonol predominantly from higher plants (e.g., *Olea europaea*)	Arrest cell cycle at S phase	[19]
Curcumin	Polyphenolic diarylheptanoid from rhizomes of Curcuma species (e.g., *Curcuma longa*)	Spheroid formation inhibition; scavenging of free radicals like ROS and RNS	[19]
Emodin	Anthraquinone derivative from medicinal plants (e.g., *Rheum palmatum* L.)	PI3K/AKT and MAPK signaling pathways	[19]
Apigenin	Flavone-type polyphenol from many herbs and vegetables (e.g., *Petroselinum crispum*)	Intrinsic apoptosis pathway	[20]
Thymol	Monoterpenoid phenol from aromatic plants (e.g., *Thymus vulgaris*)	Mitochondrial mediated apoptosis	[21]
Sulforaphane	Isothiocyanate derived from glucoraphanin in cruciferous vegetables (e.g., *Brassica oleracea*)	Cell cycle arrest and apoptosis; targets caspase 8	[22]
Kaempferol	Flavonol-type polyphenol from various plants (e.g., *Alangium salvifolium*)	Inhibition of dihydrofolate activity, thereby damaging DNA of a cancerous cell	[22]
Lupeol	Triterpenoid present in fruits and medicinal plants (e.g., *Dillenia indica*)	Induces programmed cell death (PCD)	[22]
Luteolin	Flavone-type polyphenol found in many medicinal plants (e.g., *Eclipta alba*)	Downregulating NF-κB leads to DNA damage and ultimately apoptosis	[23]
Carthamin	Quinochalcone pigment from florets of safflower (*Carthamus tinctorius*)	Caspase-3, -7, and -9 upregulation and Bcl-2 downregulation	[24]
β-Elemene	Sesquiterpene from essential oils of Curcuma species (e.g., *Curcuma wenyujin*)	Conservation/conversion of LC3-I to LC3-II	[25]
Celastrol	Triterpenoid quinone methide from *Tripterygium wilfordii*	Acts upstream of miR-101	[26]
EGCG	Catechin-type polyphenol from tea leaves (*Camellia sinensis*)	Reduces TNF-α; inhibits VCAM1, LC3A, LC3B	[27]
Honokiol	Biphenolic neolignan from Magnolia bark (*Magnolia officinalis*)	Reductions of p-PI3K, p-Akt and Ki67	[28]
Magnolol	Biphenolic neolignan from Magnolia bark (*Magnolia officinalis*)	Reductions of p-PI3K, p-Akt and Ki67	[28]

**Table 2:** Nanoparticles can be classified into various types based on various criteria [52,53].

Category	Nanoparticle Type (Size)	Key Cancer-Related Properties and Applications
Carbon-Based Nanoparticles	Fullerenes (~1–3 nm)	Inert; semiconductor/conductor; light transmission; drug delivery, photodynamic therapy
	Graphene (~5–100 nm)	High surface area; drug/gene loading; strong light absorption; imaging/therapy
	Carbon Nanotubes (CNTs, ~5–100 nm)	High tensile strength, flexible, conductive; photothermal ablation; targeted drug delivery
	Carbon Nanofibers (~50–200 nm)	Structural support; drug delivery scaffolds; biosensors for tumor detection
	Carbon Black (~50–500 nm)	High surface area; photothermal therapy; conductive carriers
Metal-Based Nanoparticles	Aluminum (~10–50 nm)	Reactive; catalytic platforms for pro-drug activation in tumors
	Iron (~10–50 nm)	Magnetic targeting; hyperthermia; MRI contrast
	Silver (~10–100 nm)	Antibacterial and anticancer coatings; photothermal therapy
	Gold (~10–100 nm)	Biocompatible; photothermal therapy, imaging, radio sensitization
	Cobalt (~10–50 nm)	Magnetic, microwave-absorbing; tumor hyperthermia (toxicity-controlled)
	Zinc (~10–50 nm)	Antibacterial/UV-filtering; tumor microenvironment modulation
Paramagnetic Metal Oxide Nanoparticles	Titanium Oxide (~10–50 nm)	Magnetic, antibacterial; photocatalysis; tumor implant coatings
	Iron Oxide (10–100 nm)	Super-paramagnetic; MRI, magnetic targeting, tumor hyperthermia
	Silicon Dioxide (20–200 nm)	Functionalizable; mesoporous silica for co-delivery of siRNA + chemo
	Zinc Oxide (~20–100 nm)	Antibacterial, UV-active; photo-therapeutics, tumor targeting
	Aluminum Oxide (~20–100 nm)	High surface area; carrier systems for drug delivery
Polymer-Based Organic Nanoparticles	Dendrimers (<10 nm)	Highly branched, surface-modifiable; targeted drug/gene delivery; enhanced tumor accumulation
	Polymeric NPs (10–1000 nm)	Biodegradable; high drug loading; controlled/sustained release; overcome solubility and drug resistance
	Polymeric Micelles (10–100 nm)	Amphiphilic block copolymer; solubilizes hydrophobic drugs; passive EPR effect; targeted delivery
	PEG-PAsp(DIP)-CA Micelle (10–100 nm)	Polymeric, pH-sensitive, paclitaxel-loaded; tumor targeting and imaging
Lipid-Based Organic Nanoparticles	Liposomes (50–100 nm)	Biocompatible vesicles; high entrapment efficiency; genes, peptides, anticancer drugs; active/passive targeting
	Solid Lipid Nanoparticles (<1 µm)	Solid lipid core; controlled release; improved stability of chemotherapeutics
	Nanostructured Lipid Carriers (~100–300 nm)	Hybrid solid–liquid core; enhanced drug loading; better release kinetics for tumors
Hybrid/Inorganic Nanoparticles	Porous Silica Nanorattle (~50–200 nm)	High surface area; docetaxel-loaded; imaging; passive EPR tumor targeting
	Gold Nanoshells (~50–100 nm)	Optical and photothermal; imaging, photothermal therapy; radiosensitization
	M13 Bacteriophage (~80–200 nm)	Biological/viral carrier; targeted delivery of anticancer payloads
	CNTs (optical imaging, ~5–100 nm)	Conductive; imaging and targeted therapy (PSMA receptor, prostate cancer)

**Table 3:** Current applications of different nanoparticles in different cancer therapies [72,73,74].

Nanoparticle Type	Drug Name	Core Composition	Key Mechanism	Clinical Indications
Lipid-Based Nanoparticles	Doxil/Caelyx	PEGylated liposomal doxorubicin	Prolonged circulation, reduced cardiotoxicity, enhanced tumor accumulation (EPR effect)	Kaposi’s sarcoma, ovarian cancer, metastatic breast cancer, multiple myeloma
	Myocet	Non-PEGylated liposomal doxorubicin	Lower cardiotoxicity than free doxorubicin; alternative for PEG-intolerant patients	Metastatic breast cancer
	Marqibo	Liposomal vincristine (sphingomyelin + cholesterol)	Extended half-life, reduced neurotoxicity, sustained release	Relapsed/refractory Philadelphia chromosome-negative ALL
	Onivyde	PEGylated liposomal irinotecan	Enhanced pharmacokinetics, improved therapeutic index	Metastatic pancreatic ductal adenocarcinoma (post-gemcitabine)
	Vyxeos	Dual-drug liposome (daunorubicin:citarabine = 1:5)	Synergistic delivery, prolonged bone marrow exposure	High-risk acute myeloid leukemia (AML)
	Lipusu (China)	Non-PEGylated paclitaxel liposome	Improved solubility, reduced solvent-associated toxicity	Breast, ovarian, and non-small cell lung cancer
	Genexol (Korea, India)	Polymeric paclitaxel micelle formulation	Enhances solubility, reduced systemic toxicity	Breast cancer, non–small cell lung cancer
	Nanoxel (Korea, India)	Polymer-based paclitaxel nanoparticles	Improved tumor penetration, solvent-free formulation	Metastatic breast cancer, NSCLC, Kaposi’s sarcoma
	Bevetex (India)	Polymer–lipid hybrid paclitaxel nanoparticle	Improved delivery efficiency, reduced toxicity	Ovarian, breast, bladder cancers, Kaposi’s sarcoma
Protein-Based Nanoparticles	Oncaspar	PEGylated L-asparaginase (enzyme nanoparticle)	Depletes asparagine; PEGylation reduces immune reaction	Acute lymphoblastic leukemia (ALL)
	Ontak	Diphtheria toxin–IL-2 fusion protein	Targeted delivery to IL-2 receptor cancer cells; inhibits protein synthesis	Cutaneous T-cell lymphoma
	Eligard	Nanoparticle depot of leuprorelin	Continuous hormone suppression (lowers testosterone/estrogen)	Prostate cancer, breast cancer, endometriosis
Albumin-Based Nanoparticles (Nab)	Abraxane/Pazenir	Albumin-bound paclitaxel nanoparticles	gp60-mediated transcytosis + SPARC-mediated tumor uptake; solvent-free	Breast cancer, NSCLC, pancreatic cancer
Antibody–Drug Conjugate (ADC)	Kadcyla (T-DM1)	Trastuzumab linked to DM1 cytotoxin	HER2-targeted delivery; internalization and intracellular drug release	HER2-positive breast cancer
Metal-Based Nanoparticles	NanoTherm	Amino-silane–coated iron oxide nanoparticles (15 nm)	Magnetic field–induced hyperthermia; tumor-localized heating	Glioblastoma multiforme, prostate cancer

**Table 4:** Patent landscape of different nanomedicine and their patent ID [99,100].

Patent ID	Description
Patent IN202241000705 (2022)	The patent describes novel drug compositions that may be utilized in the treatment of lung cancer, including non-small cell lung carcinoma (NSCLC), that consist of Astragalus, cisplatin, and vinorelbine.
Patent IN202141046188 (2021)	The study reports a novel approach of anticancer activity enhancement by quercetin-loaded TPGS nanosuspension with effective induction of apoptosis in drug-resistant MCF-7 human breast cancer cells.
Patent IN202021048696 (2020)	The invention reports a silver nanoparticle formulation of cytotoxic herbal extract derived from *Brassica oleracea* L. that targets mammary carcinoma, cervical cancer, and hepatocarcinoma cells through the induction of G2/M-phase cell cycle arrest.
Patent IN202041023550 (2020)	A novel, non-invasive synergistic nanoformulation of *Tridax procumbens* L., *Curcuma longa* L., and *Trachyspermum ammi* (L.) Sprague was developed for the prevention and treatment of human lung cancer with great efficacy, possessing anticancer, cytotoxic, and wound-healing properties.
Patent IN202041025649 (2020)	This polyherbal medicine, made up of nine medicinal plant components like *Plumbago zeylanica* L., *Zingiber officinale* Roscoe., and *Terminalia chebula* Retz., has shown excellent activity in the prevention and treatment of gastric and colon cancer.
Patent US20170258929 (2017)	It is a method of using a conjugate of GnRH and curcumin, by itself or in combination with 2′,2′-difluoro-2′-deoxycytidine, as an effective therapy in the treatment of pancreatic cancer.
Patent EP3144006 (2017)	The current invention pertains to a liposomal curcumin formulation that effectively eliminates QT prolongation and is therefore a promising combination therapy in conjunction with chemotherapeutic medications in the treatment of glioblastoma.
Patent US20170035701 (2017)	A novel preparation method has been developed for a stabilized high drug load nanocarrier in which curcumin, resveratrol, honokiol, and magnolol are encapsulated within a lipid shell micellar core for enhanced therapeutic activity against brain, liver, and skin cancer.
Patent US20170189343 (2017)	The current invention is associated with a nano drug carrier for targeted drug delivery to tumors based on a depolymerization-polymerization mechanism of human ferritin for encapsulating various anticancer bioactive compounds for the treatment of hematological cancers.
Patent US20170224636 (2017)	A curcumin–sophorolipid complex nano-encapsulated form has been prepared for enhancing the bioavailability of curcumin in sophorolipid acidic environment, exhibiting breast cancer therapeutic potential.
Patent US20160287533 (2016)	This curcumin bioavailability-enhancing composition, which is prepared using curcumin, resveratrol, and catechin derivatives, enhances the properties of curcumin and is used for treating uncontrollable cell growth, especially for cervical cancer and precancerous cervical lesions.
Patent US20160287706 (2016)	A new left helical 3D nanocage structure has been designed as a highly efficient drug carrier for cell delivery and is particularly suited for delivering genistein and other active herbal molecules for the treatment of various cancers.
Patent W02016014337 (2016)	The current invention reveals a nanoemulsion-based drug delivery system for enhancing the bioavailability and therapeutic efficacy of curcumin in the treatment of colorectal cancer.
Patent US20150314006 (2015)	A novel particulate drug delivery system was developed on the basis of a drug-polymer or oligomer conjugate containing genistein-like derivatives with one hydroxyl group and a thiol group, which is a promising therapeutic system against prostate cancer.

## Data Availability

No new data were created or analyzed in this study. Data sharing is not applicable to this article.

## Abbreviations

Abbreviation	Full form
EPR	Enhanced permeability and retention
TME	Tumor microenvironment
ECM	Extracellular matrix
CAF(s)	Cancer-associated fibroblast(s)
CSC(s)	Cancer stem cell(s)
DC(s)	Dendritic cell(s)
CTL(s)	Cytotoxic T lymphocyte(s)
TAM(s)	Tumor-associated macrophage(s)
MDSC(s)	Myeloid-derived suppressor cell(s)
MDR	Multidrug resistance
NP/NPs	Nanoparticle(s)
SPION(s)	Superparamagnetic iron oxide nanoparticle(s)
SLN(s)	Solid lipid nanoparticle(s)
MAPK	Mitogen-activated protein kinase
PAMT	PI3K/Akt/mTOR
PI3K	Phosphoinositide 3-kinase
mTOR	Mechanistic target of rapamycin
ROS	Reactive oxygen species
RNS	Reactive nitrogen species
BAX	BCL-2–associated X protein
Bcl-2	B-cell lymphoma 2
ERK	Extracellular signal-regulated kinase
CaMKII	Calcium/calmodulin-dependent protein kinase II
CDK8	Cyclin-dependent kinase 8
EMT	Epithelial–mesenchymal transition
OXPHOS	Oxidative phosphorylation
SELEX	Systematic Evolution of Ligands by Exponential enrichment
SNA(s)	Spherical nucleic acid(s)
GBM	Glioblastoma multiforme
PSA	Prostate-specific antigen
PLGA	Poly(D,L-lactide-co-glycolide)
